# A Case of Acquired Hemophilia A in a Patient with Exposure to COVID-19

**DOI:** 10.1155/2022/9494249

**Published:** 2022-03-22

**Authors:** Jorge D. Guerra, Jasmine Gowarty, Jordan Buess, James Mason, Kathleen Halka

**Affiliations:** ^1^Department of Internal Medicine, Baylor Scott & White Medical Center, Texas A&M University College of Medicine, Temple, TX, USA; ^2^Department of Hematology and Medical Oncology, Baylor Scott & White Medical Center, Texas A&M University College of Medicine, Temple, TX, USA

## Abstract

Acquired Hemophilia A (AHA) is a very rare autoimmune condition involving immune-mediated depletion of Factor VIII, resulting in spontaneous hemorrhage. Failure to recognize AHA as a possible etiology of hemorrhage can result in delayed diagnosis and treatment. The COVID-19 pandemic has given rise to several hematologic conditions and complications, with a rare manifestation being Acquired Hemophilia A (AHA). An interesting case of AHA following SARS-CoV-2 infection is described, along with the treatment approach and a brief review of several cases describing AHA in association with COVID-19.

## 1. Introduction

Acquired Factor VIII deficiency, also referred to as Acquired Hemophilia A (AHA), is a rare condition that entails antibody-mediated depletion of Factor VIII, resulting in spontaneous hemorrhage in patients without a history of bleeding [1, 2]. Traditionally, AHA is found in elderly men or young women and is oftentimes associated with pregnancy or the postpartum period [1]. Data trends have suggested that approximately 50% of AHA cases are classified as idiopathic while the remaining half are associated with other autoimmune disorders, malignancies, infections, drugs, and certain dermatologic disorders [2]. Although hemarthrosis is classically associated with hemophilia A, there is a higher prevalence of hemorrhage from the soft tissues, abdomen, and retroperitoneum in acquired hemophilia as compared to congenital hemophilia [3]. Therefore, bleeding associated with AHA can be severe and life threatening as described in this case of *de novo* AHA occurring in the setting of recent infection with SARS-CoV-2, the virus causing COVID-19 in humans.

## 2. Case Report

A 74-year-old female with a history of recent infection with SARS-CoV-2, hypertension, and fibromyalgia presented as a transfer from an outlying facility with a history of gross hematuria beginning approximately four weeks after recovery from what was classified an NIH mild-grade COVID-19 illness [4]. Computed tomography (CT) scanning showed no findings concerning for malignancy. Prior to transfer, she underwent evaluation with urology to include cystoscopy, which identified bleeding from the right ureteral orifice, as well as retrograde pyelography and ureteroscopy, identifying a large clot in the right renal pelvis. She additionally required laser hemostasis treatment for renal calyceal bleeding and placement of ureteral stents. During her stay at the outside hospital, she received four units of blood for hemoglobin of less than 7.0 g/dL. Due to ongoing bleeding despite procedural interventions, she was transferred for higher level of care. Factor levels had been ordered prior to transfer but were send-out labs and thus not available for several days.

Her vitals were stable on arrival after transfer. Physical exam was significant for right costovertebral angle tenderness to palpation. Labs demonstrated normocytic anemia with hemoglobin of 10.1 g/dL (reference: 12–16 g/dL), elevated white blood cell count of 14.1 × 10^9^/L (reference: 4.8–10.8 × 10^9^/L), normal platelet count of 351 × 10^9^/L (reference: 150–450 × 10^9^/L), prolonged activated partial-thromboplastin time (aPTT) of 80.1 seconds (reference: 25.1–36.5 sec), and normal prothrombin time (PT) of 12.5 seconds (reference: 12.2–15.3 sec). Additional studies revealed creatinine of 1.73 (reference: 0.50–1.30 mg/dL) in this patient with previously normal renal function. Urinalysis and urine cultures did not reveal evidence of an infection. She was evaluated by the local urology service with additional procedures, including electrode fulguration of bleeding areas with placement of a right percutaneous nephrostomy drain. Interventional Radiology (IR) was subsequently consulted and performed renal angiography with embolization of an arteriovenous malformation in the right upper pole along with coiling of a nephrostomy tract pseudoaneurysm. Due to persistent bleeding after addressing these anatomical and procedure-related issues, Hematology/Oncology was consulted for suspicion of a bleeding disorder. Lab results from the outside facility became available at that time, demonstrating Factor VIII level of 3%. Based on this result and on mixing studies suggestive of inhibitor presence, the patient was empirically treated with factor eight inhibitor bypass agent (FEIBA) and prednisone. Repeat studies requested by the hematology service demonstrated Factor VIII activity of 0% with the presence of Factor VIII inhibitor measured at 48 Bethesda units (BU), confirming the diagnosis of AHA. The remaining factor levels were normal, without any other inhibitors identified. Other malignant, drug-induced, and infectious etiologies were investigated and results were ultimately unrevealing.

Due to ongoing bleeding, treatment escalations were required, including NovoSeven (a recombinant human coagulation Factor VIIa), cyclophosphamide, and ultimately a single dose of rituximab. Hematuria had nearly resolved by 48 hours after rituximab administration and had fully resolved by 72 hours, allowing discharge from the hospital with continued prednisone and cyclophosphamide use in the outpatient setting and planned rituximab treatments weekly to complete four infusions. Repeat labs one week after discharge revealed a Factor VIII activity of 18% with a reduced inhibitor level of 9.2 BU. Cyclophosphamide was stopped at this time due to neutropenia. Labs two weeks thereafter revealed no evidence of inhibitor, with a Factor VIII activity of 43%. After two additional weeks and completion of rituximab infusions, she achieved a normal Factor VIII activity level of 71%, with inhibitor no longer identified and with normal hemoglobin of 13.6 g/dL. She slowly weaned prednisone over the course of several weeks and has not had any recurrence of AHA.

## 3. Discussion

AHA is a rare etiology of life-threatening hemorrhage mediated by autoantibodies directed against Factor VIII [1, 2]. Although no predisposing disorder is identified in approximately half of cases, an awareness of associated conditions may lead to earlier diagnosis in patients presenting with hemorrhage and concomitant diagnoses associated with AHA [2]. Some of these established diagnoses associated with AHA include other viral illnesses, such as human immunodeficiency virus (HIV), Hepatitis B and C viruses, and Epstein-Barr virus (EBV), along with various autoimmune conditions, such as autoimmune hemolytic anemia (AIHA)—a better understood autoimmune hematologic disease [1, 5–7]. Several studies have demonstrated a close relationship between AIHA and T-helper type 2 (Th2) cell response—particularly T-helper 17 (Th17) cell and regulatory T-cell (Treg) immune dysregulation [6]. An increased number of Th17 cells in discordance are thought to be a contributing pathway of AIHA development and, with increasing levels, a predictive model correlating with AIHA disease activity [6]. Other studies of viral-induced immune dysregulation, specifically related to influenza and SARS-CoV-2, have demonstrated such imbalance in both Th cells and suppressor T-cell activity. Ongoing investigative efforts suggest that severe manifestations of COVID-19, as well as associated complications, could be related to these findings [8].

A review by Barcellini et al. discusses autoimmune complications in hematologic neoplasms, including development of AHA, AIHA, immune thrombocytopenia (ITP), systemic lupus erythematosus (SLE), rheumatoid arthritis (RA), and antiphospholipid syndrome (APS) [7]. Autoimmunity is described as complex, related to genetic and environmental factors, and primary immunodeficiencies leading to blunted, imbalanced immune response. Patients with this demographic have additional components of immunosuppression, further worsening immune dysregulation. Treg imbalance associated with miRNA dysregulation is one such component of immune dysregulation thought to be related to the development of various autoimmune conditions such as AHA [7]. Virus-derived miRNAs, as established in EBV, have also been implied in the pathogenesis of autoimmunity. Other noteworthy mechanisms that can induce autoimmunity include molecular mimicry, leading to T- and B-lymphocyte activation, clonality, and discordance. Such mechanisms are utilized by several viruses, bacteria, and parasites [7].

A search of the literature revealed three other cases of AHA attributed to COVID-19. The first case involved an 83-year-old female patient who presented with diffuse bruising a week after recovering from NIH mild-grade COVID-19. Treatment involved administration of both prednisone and rituximab [9]. The second case was of a 66-year-old male who had AHA *re-activation* causing hematoma in the trunk, which was identified in 2020 during admission for COVID-19 pneumonia. He had been previously diagnosed and treated for AHA in 2011, which was managed then with intravenous recombinant activated Factor VII, prednisone, and once-weekly cyclophosphamide for a total of 4 weeks. Remission was achieved at day 21. On relapse, he was managed with the same antihemorrhagic and immunosuppressive treatment with remission achieved at day 20. He additionally received lopinavir-ritonavir and supplemental oxygen for treatment of COVID-19 pneumonia [2]. The third case described a 65-year-old man who presented with symptoms of severe subcutaneous bleeding in his arm leading to fasciotomy and mass transfusion protocol. Work-up revealed elevated PTT, decreased Factor VIII, and elevated Factor VIII inhibitor levels. He had elevated SARS-CoV-2 antibody levels with a negative SARS-CoV-2 PCR test. It was concluded that the patient's new findings may have been linked to previous asymptomatic COVID-19. He was treated with a combination of NovoSeven, stress-dose steroids (with taper), a 4-week course of weekly rituximab, followed by a cyclophosphamide taper. Due to persistence of elevated inhibitor levels, the patient was also treated with two doses of FEIBA. Bleeding resolved and Factor VIII normalization was documented at 3-week follow-up [10].

While the pathophysiology of AHA remains unclear and the link with SARS-CoV-2 even more so, there is growing evidence associating COVID-19 with hematological and nonhematological autoimmune diseases including cold agglutinin autoimmune hemolytic anemia, thrombotic thrombocytopenic purpura, Guillain–Barré syndrome, and immune thrombocytopenic purpura [11–14]. Given these associations, as well as known associations of other viral infections with AHA, it is prudent to consider COVID-19-associated AHA in patients with hemorrhage occurring in the setting of recent or current SARS-CoV-2 infection.

## 4. Conclusion

To our knowledge, this is the third reported case in the literature of *de novo* AHA associated with SARS-CoV-2 infection and the first reported case of a spontaneous urological bleed due to AHA. This relationship was established given the absence of prior history of spontaneous bleeding, autoimmune disease, malignancy, or other offending agents or infections and the known dysregulation of the hemostatic system by SARS-CoV-2 [15]. Due to the severity and life-threatening nature of this disease, a high suspicion for AHA must be maintained in patients presenting with unexplained or worsening hemorrhage after exposure to SARS-CoV-2.

## Data Availability

The data used to support the findings of this study are included within the article.
